# Intestinal Parasite Prevalence in an Area of Ethiopia after Implementing the SAFE Strategy, Enhanced Outreach Services, and Health Extension Program

**DOI:** 10.1371/journal.pntd.0002223

**Published:** 2013-06-06

**Authors:** Jonathan D. King, Tekola Endeshaw, Elisabeth Escher, Genetu Alemtaye, Sileabatt Melaku, Woyneshet Gelaye, Abebe Worku, Mitku Adugna, Berhanu Melak, Tesfaye Teferi, Mulat Zerihun, Demelash Gesese, Zerihun Tadesse, Aryc W. Mosher, Peter Odermatt, Jürg Utzinger, Hanspeter Marti, Jeremiah Ngondi, Donald R. Hopkins, Paul M. Emerson

**Affiliations:** 1 The Carter Center, Atlanta, Georgia, United States of America; 2 Department of Epidemiology and Public Health, Swiss Tropical and Public Health Institute, Basel, Switzerland; 3 University of Basel, Basel, Switzerland; 4 The Carter Center, Addis Ababa, Ethiopia; 5 Department of Medical Services and Diagnostic, Swiss Tropical and Public Health Institute, Basel, Switzerland; 6 Amhara Regional Research Laboratory, Amhara National Regional State Health Bureau, Bahir Dar, Ethiopia; 7 South Gondar Zonal Health Department, Amhara National Regional State Health Bureau, Debre Tabor, Ethiopia; 8 Department of Public Health and Primary Care, Institute of Public Health, University of Cambridge, Cambridge, United Kingdom; University of Queensland, Australia

## Abstract

**Background:**

The SAFE strategy aims to reduce transmission of *Chlamydia trachomatis* through antibiotics, improved hygiene, and sanitation. We integrated assessment of intestinal parasites into large-scale trachoma impact surveys to determine whether documented environmental improvements promoted by a trachoma program had collateral impact on intestinal parasites.

**Methodology:**

We surveyed 99 communities for both trachoma and intestinal parasites (soil-transmitted helminths, *Schistosoma mansoni*, and intestinal protozoa) in South Gondar, Ethiopia. One child aged 2–15 years per household was randomly selected to provide a stool sample of which about 1 g was fixed in sodium acetate-acetic acid-formalin, concentrated with ether, and examined under a microscope by experienced laboratory technicians.

**Principal Findings:**

A total of 2,338 stool specimens were provided, processed, and linked to survey data from 2,657 randomly selected children (88% response). The zonal-level prevalence of *Ascaris lumbricoides*, hookworm, and *Trichuris trichiura* was 9.9% (95% confidence interval (CI) 7.2–12.7%), 9.7% (5.9–13.4%), and 2.6% (1.6–3.7%), respectively. The prevalence of *S. mansoni* was 2.9% (95% CI 0.2–5.5%) but infection was highly focal (range by community from 0–52.4%). The prevalence of any of these helminth infections was 24.2% (95% CI 17.6–30.9%) compared to 48.5% as found in a previous study in 1995 using the Kato-Katz technique. The pathogenic intestinal protozoa *Giardia intestinalis* and *Entamoeba histolytica/E. dispar* were found in 23.0% (95% CI 20.3–25.6%) and 11.1% (95% CI 8.9–13.2%) of the surveyed children, respectively. We found statistically significant increases in household latrine ownership, use of an improved water source, access to water, and face washing behavior over the past 7 years.

**Conclusions:**

Improvements in hygiene and sanitation promoted both by the SAFE strategy for trachoma and health extension program combined with preventive chemotherapy during enhanced outreach services are plausible explanations for the changing patterns of intestinal parasite prevalence. The extent of intestinal protozoa infections suggests poor water quality or unsanitary water collection and storage practices and warrants targeted intervention.

## Author Summary

Part of the SAFE strategy (surgery, antibiotics, facial cleanliness, and environmental improvement) to eliminate blinding trachoma involves improving access to, and use of, water and sanitation. We combined the assessment of parasitic worm and intestinal protozoa infections with surveys of trachoma in an area of Ethiopia where the SAFE strategy, together with enhanced outreach services and the health extension program, had been implemented for more than 5 years. We compared our findings with results from a survey conducted in the mid-1990s. We documented significant increases in household access and use of latrines and clean water: the F and E components of the SAFE strategy as promoted by the health extension program. We found considerably lower levels of parasitic worm infections than those reported previously. Moreover, we documented, for the first time in this zone, pathogenic intestinal protozoa infections, which indicate poor water quality and unhygienic water collection and storage practices in the communities surveyed. A plausible hypothesis for the decline in parasitic worm infections might be the combined impact of ongoing simultaneous health programs: SAFE strategy for trachoma control alongside the health extension program and regular deworming of preschool-aged children.

## Introduction

An integrated strategy of surgery, antibiotics, facial cleanliness, and environmental improvement – the SAFE strategy in short – is recommended to eliminate blinding trachoma in endemic countries by the year 2020 [1]. The F and E components aim to reduce the transmission of *Chlamydia trachomatis* via flies, fingers, and fomites within the community [2]. Face washing is promoted specifically to keep faces free of infectious ocular and nasal discharge, and make them less attractive to eye-seeking flies. The construction and use of latrines are promoted as a form of fly control to reduce fly-to-eye contact [2], [3]. Improved accessibility to clean water is also promoted, but whether or not water is used for hygiene is more important than absolute access to clean water in trachoma prevention. Where water is not readily accessible, household use of a limited supply of water may not be prioritized for bathing [4]–[6]. These aims of the F and E components go beyond trachoma control and align with other major initiatives, such as the WASH program of UNICEF and Millennium Development Goal (MDG) 7c which, by 2015, aim to provide access to clean water and sanitation to all children and to reduce by half the proportion of households without access to basic sanitation [7], [8].

Improved hygiene, sanitation, and water have a positive and sustained impact on several diseases, including many of the neglected tropical diseases [9], [10]. Trachoma was eliminated from the United States of America (USA) primarily through sustained social and economic development [11]. The Rockefeller Foundation noted the pivotal role sanitation played in the elimination of hookworm in the southern parts of the USA some 100 years ago [12]. Improving water supply and sanitation have been recommended after noting the reduction in the prevalence and incidence of parasitic worms such as dracunculiasis and soil-transmitted helminthiasis, and diarrhea as well as an increase in child survival [13]. A systematic review and meta-analysis of studies reporting the effects of sanitation on soil-transmitted helminth infections (*Ascaris lumbricoides*, *Trichuris trichiura*, and hookworm) found that having access and using sanitation was associated with an approximately 50% lower odds of any soil-transmitted helminth infection even after accounting for random effects between studies [10].

These are assumed ancillary benefits of the activities promoted by the F and E components of the SAFE strategy, yet these have not been fully documented in the context of an ongoing trachoma control program. The purpose of this study was to determine the prevalence of intestinal parasites (soil-transmitted helminths, *Schistosoma mansoni*, and intestinal protozoa) among children aged 2–15 years to complement a large trachoma impact survey in 2011. The data also allowed to study changing patterns of parasitic worm infections in the school-aged population by comparing our findings to those obtained in a survey conducted in the mid-1990s [14]. We aimed also to determine whether improvements in household-level access to water and sanitation have occurred in this zone of the Amhara National Regional state in Ethiopia after the SAFE strategy had been fully implemented for at least 5 years.

## Methods

### Ethics Statement

The study protocol was reviewed and approved by the ethical review committee of the Amhara National Regional State Health Bureau. Additionally, the study activities, including oral consent, were approved by Emory University Institutional Review Board (protocol no. 079-2006). According to the principles of the Helsinki Declaration, informed consent for the interview and for stool examinations was sought. Due to the high rate of illiteracy, oral informed consent was obtained from the parent or guardian and recorded in the electronic survey form. Additionally verbal assent was obtained from children aged 7 years and above and also recorded in the electronic survey form. Each selected child, regardless of participation, was offered a single dose of albendazole (400 mg) during the household visit.

### Study Area and Population

The study was conducted in South Gondar zone of the Amhara regional state of Ethiopia in the rainy season from late June to early August 2011, covering all 10 rural *woredas* (districts) in the zone. The two semi-urban *woredas* excluded from the survey were the zonal capital, Debra Tabor Town and Woreta Town. The total population of South Gondar is approximately 2.05 million people with 1.86 million living in the 10 surveyed *woredas* [15]. The elevation in the zone ranges from 600 to >4,000 m above sea level and is geographically diverse with areas of lake shore, lowlands, highland plateaus, rugged mountain peaks, and valleys. People are primarily engaged in subsistence agriculture; rice in the lake shore areas, wheat and teff in hill and mountain sides, and animal husbandry in all areas.

### Sample Size and Sampling Methodology

We assumed a null hypothesis of no change in the prevalence of infection with any of the following helminths, *A. lumbricoides*, *T. trichiura*, hookworm, and *S. mansoni*, as assessed in a cross-sectional survey of school-aged children of South Gondar in 1995, when it was estimated at 49% [14]. In order to detect at least a 20% decline in prevalence (from 49% to 29%) at the 5% level of significance and power of 90%, stool specimens from 800 school-aged children (7–15 years) needed to be examined assuming a design effect of 4 for the multi-stage cluster random sampling methodology implemented. We oversampled and included children 2–6 years of age to assess the prevalence of helminths and intestinal protozoa infections in this age group currently receiving preventive chemotherapy with albendazole during biannual campaigns known as enhanced outreach services (EOS) [16]. Additionally, given the focal nature of some helminth infections (e.g. *S. mansoni*), we aimed to select a geographically representative sample from each of 10 *woredas* by systematically selecting 10 *gotts* (communities) from a random starting *gott* from *woreda*-specific lists arranged geographically. In each *gott*, one child aged 2–15 years was selected randomly in each of 30 surveyed households and asked to provide a single stool sample. Households were selected randomly using a modified segmentation design, and children were selected randomly by an electronic data collection device (see below) after enumerating all residents, both present and absent, of the selected household [17].

### Survey Tool and Stool Sample Processing

Household sanitation characteristics were determined and recorded at each consenting household by observing the presence of a used latrine and hand washing container noted with or without water. A used latrine was defined as directly observing feces in the pit with the use of a torch if needed. The head of household or adult representative was interviewed about access to, and use of, water. The selected child and the parent/guardian were shown the albendazole tablets distributed during EOS campaigns and were asked whether the child had received and taken the drug.

Using small portable scales to measure submitted stool samples, field teams recorded the exact weight and fixed approximately 1 g of stool in 10 ml of sodium acetate-acetic acid-formalin (SAF) solution [18]. Fixed specimens were labeled with unique identification numbers (IDs), transferred to a central storage area at room temperature, and shielded from direct sunlight [19]. Upon completion of the field data collection, all specimens were processed at the Amhara Regional Research Laboratory using an ether-concentration method that has shown good reliability among European reference laboratories [20]. The entire sediment was assessed systematically for helminth eggs and intestinal protozoa cysts. For helminths, the number of eggs identified were counted and recorded (1 up to 100 eggs). Counting stopped above 100 eggs and was recorded as 100+. The frequency of intestinal protozoa cysts were recorded as none, rare (1–5 parasites per slide), frequent (1 parasite per observing field), and very frequent (>1 parasite per observing field).

### Training and Quality Control

Prior to the field data collection, teams participated in a 7-day, applied training for data collectors (health facility-based laboratory technicians), which consisted of classroom instruction and field practice where the protocol and data collection tools were refined, and adapted to the local context. Technicians processing the stool specimens were trained in the ether-concentration method, reading slides, and identification of parasites at species level. Every tenth negative specimen and every specimen where a helminth was identified by a technician was reexamined by a senior laboratory technician.

### Data Management and Statistical Analysis

Survey data were collected electronically using tablet computers operating on the Android (Google Inc.; Mountain View, CA, USA) platform, and were linked to results of processed specimens via the unique ID on each specimen. Laboratory results were recorded on paper forms by technicians and then double-entered in Microsoft Access by separate entry clerks, compared for discordance, and corrected with the original hard-copy.

Data were analyzed using SAS version 9.3 (SAS Institute Inc.; Cary, NC, USA). Selection probabilities were calculated and used to weight the data in the analysis. Additionally, the variance of the estimates was adjusted to account for clustering. To measure differences in household-level access to, and use of, water and sanitation, the current survey data were compared to household survey data collected in 2000 and 2003 prior to any interventions, and 2006 after interventions in only three of 13 districts. All surveys were conducted by the Amhara National Regional State Health Bureau and The Carter Center using the same cluster, randomized survey methodology [21], [22].

## Results

### Participation and Final Study Sample

**Figure 1:**
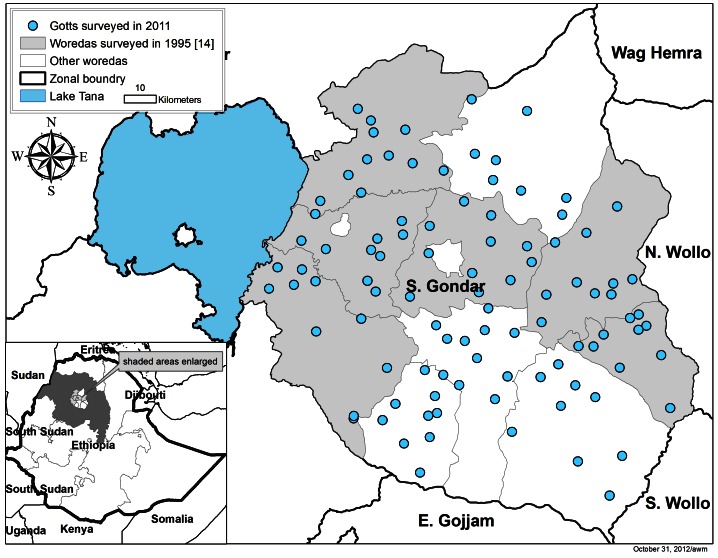
Location of *gotts* surveyed for both intestinal parasites and trachoma in South Gondar, Amhara Region, Ethiopia in mid-2011.

**Figure 2:**
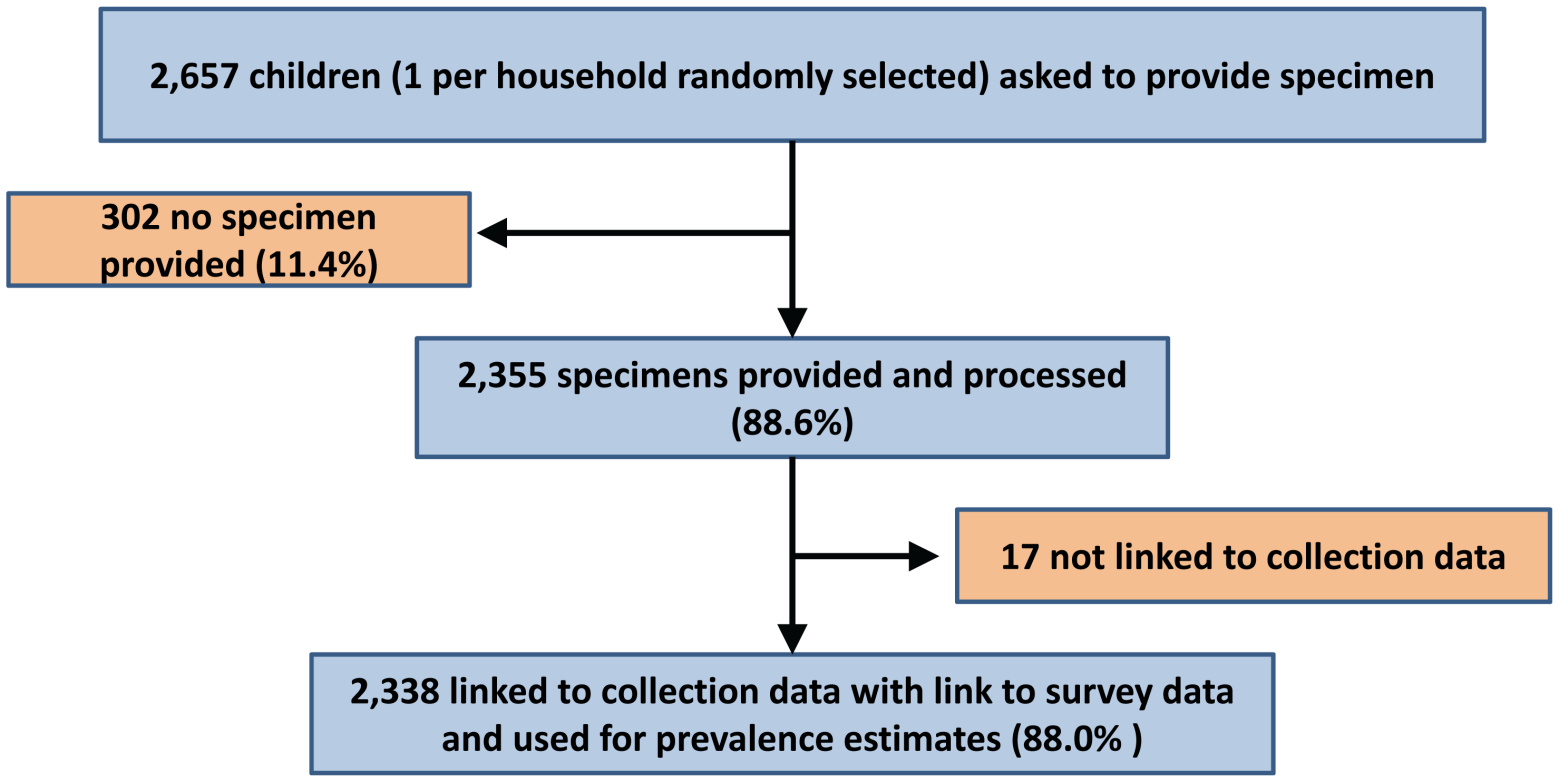
Flow chart of survey sample.

Figure 1 shows the geographical distribution of the 99 surveyed communities across 10 *woredas* as well as the *woredas* where schools were surveyed in 1995. A total of 2,355 stool samples were provided and processed from 2,657 randomly selected children aged 2–15 years in surveyed households (88.6%). Figure 2 shows the resulting sample sizes used in the analysis. Mean age of children submitting samples was 6.8 years (standard deviation (SD) 3.6 years) and 48.0% of specimens were from boys.

### Helminth and Intestinal Protozoa Infection

**Figure 3:**
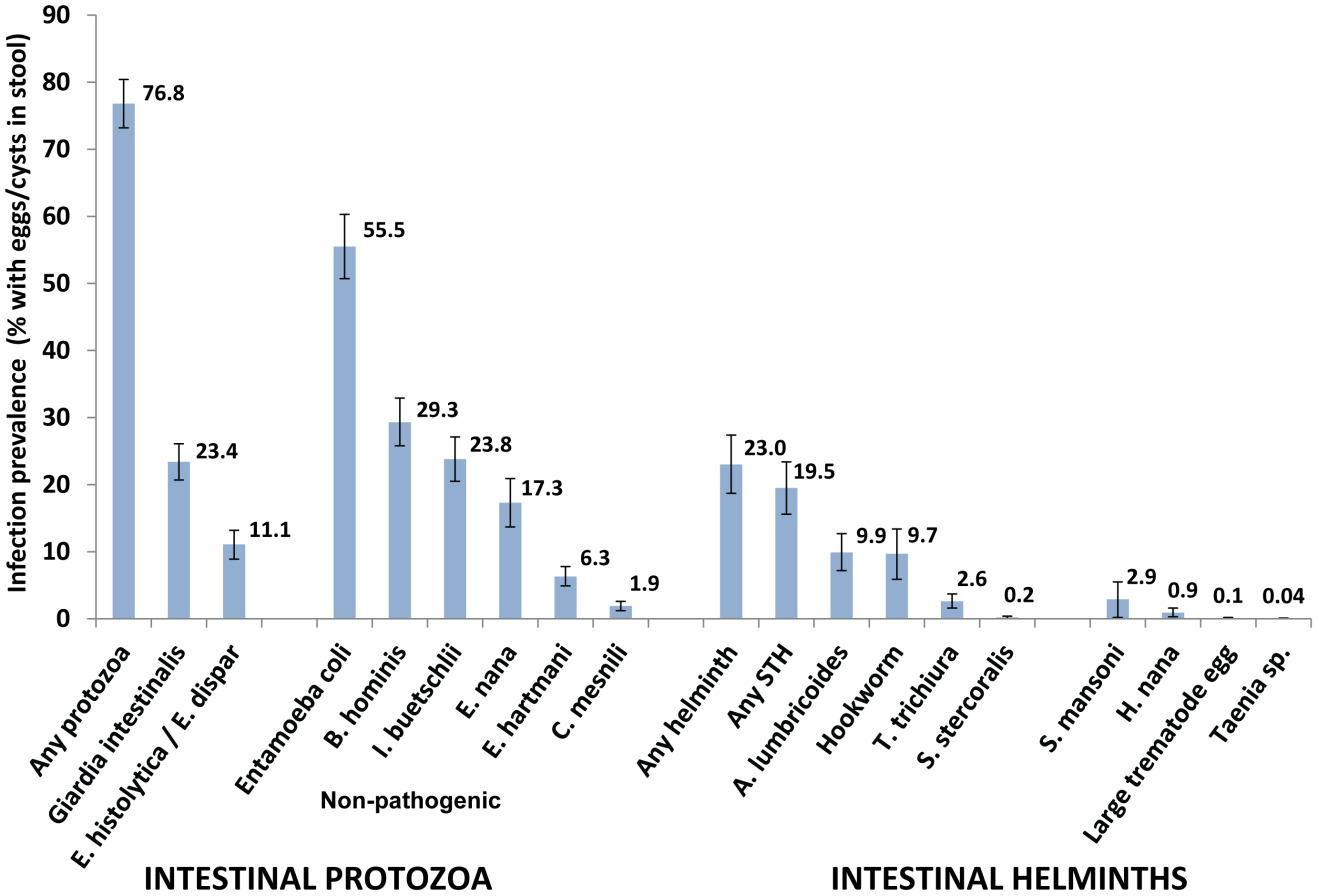
Prevalence of intestinal parasites among children aged 2–15 years in South Gondar, Amhara, Ethiopia in mid-2011.

Helminth eggs and intestinal protozoa cysts were examined in 2,338 processed stool specimens (Figure 3). The prevalence of any intestinal protozoa infection (76.8%; 95% confidence interval (CI) 73.2–80.4%) was higher than the prevalence of any helminth infection (23.0%; 95% CI 18.7–27.4%) in this study. The prevalence of the two intestinal protozoa *Giardia intestinalis* and *Entamoeba histolytica/E. dispar* was 23.4% (95% CI 20.7–26.1%) and 11.1% (95% CI 8.9–13.2%), respectively. The prevalence of infections where cysts of any intestinal protozoa were identified as very frequent was 11.0% (95% CI 9.5–12.6%) and the majority of these were *Entamoeba coli*.

Among the helminths, *A. lumbricoides* and hookworm were the most frequently observed with point prevalence of 9.9% (95% CI 7.2–12.7%) and 9.7% (95% CI 5.9–13.4%), respectively. Including *T. trichiura* and *Strongyloides stecoralis* (larvae), the prevalence of infection with any of these soil-transmitted helminths was 19.5% (95% CI 15.6–23.4%). At the *woreda* level, the prevalence of any soil-transmitted helminths ranged from 7.9% to 34.6% (Table 1). The prevalence of any soil-transmitted helminth among preschool-aged children (Table 2) was 17.4% (95% CI 13.0–21.7%) and was not significantly different from prevalence among school-aged children (21.4%, 95% CI 16.5–26.4%; p = 0.106). The prevalence of *S. mansoni* was 2.9% (95% CI 0.2–5.5%; range by *woreda* 0–12.5%). In surveyed *gotts*, the proportion of children with *S. mansoni* ranged from nil to 52.4%.

**Table 1:** Prevalence* of soil-transmitted helminths (STH) and *Schistosoma mansoni* among children aged 2–15 years.

*Woreda*	Any STH			*A. lumbricoides*			*T. trichiura*			Hookworm			*S. mansoni*		
	(%)	95% CI		(%)	95% CI		(%)	95% CI		(%)	95% CI		(%)	95% CI	
West estie (N = 246)	34.6	22.0	47.2	22.2	11.9	32.5	5.8	0.4	11.2	13.4	1.1	25.7	1.3	0.0	3.2
Dera (N = 248)	33.0	25.0	41.1	3.6	0.0	7.4	2.0	0.0	5.6	30.5	21.6	39.4	3.3	0.0	7.3
Fogera (N = 227)	26.5	13.0	39.9	11.3	0.6	22.0	3.1	0.0	6.1	19.3	7.4	31.1	12.5	0.0	32.8
East Estie (N = 212)	23.5	13.2	33.8	18.4	7.9	29.0	4.5	1.6	7.4	2.9	0.0	6.2	0.0	.	.
Ebinat (N = 235)	17.5	7.0	28.0	11.1	0.1	22.1	1.8	0.2	3.5	6.3	1.1	11.5	0.2	0.0	0.5
Libokemkem (N = 209)	17.2	12.4	21.9	9.7	6.6	12.8	3.7	1.3	6.0	6.5	0.9	12.0	7.7	0.7	14.7
Farta (N = 249)	15.2	5.1	25.2	7.2	0.6	13.9	3.6	0.3	7.0	6.4	0.0	15.7	0.5	0.0	1.4
Lay gayint (N = 243)	10.9	1.5	20.2	9.3	0.9	17.6	0.0	.	.	1.8	0.0	3.8	0.0	.	.
Simada (N = 241)	8.2	5.1	11.3	5.2	2.5	8.0	1.8	0.3	3.2	2.0	0.2	3.8	0.2	0.0	0.5
Tach gayint (N = 228)	7.9	0.1	15.7	7.3	0.0	14.7	0.7	0.0	2.1	0.2	0.0	0.7	0.7	0.0	2.0
**Zone (N = 2,338)**	19.5	15.6	23.4	9.9	7.2	12.7	2.6	1.6	3.7	9.7	5.9	13.4	2.9	0.2	5.5

Results from a cross-sectional survey in South Gondar zone, Amhara Regional State, Ethiopia in 2011.

* Estimates weighted according to selection probabilities and adjusted for correlation in the data due to clustering.

CI, confidence interval.

**Table 2:** Prevalence* of any soil-transmitted helminths (STH) among preschool-aged and school-aged children.

	Preschool-aged children (2–6 years)				School-aged children (7–15 years)			
	Any STH				Any STH			
*Woreda*	n	%	95% CI		n	%	95% CI	
West Estie	150	31.9	17.4	46.4	91	38.0	25.3	50.7
Dera	133	33.8	22.9	44.6	111	31.4	13.8	49.0
Fogera	112	23.4	7.0	39.8	112	28.9	14.1	43.7
East Estie	118	24.9	11.8	38.0	91	22.5	11.5	33.5
Ebinat	120	12.6	2.5	22.6	110	21.9	10.9	32.8
Libokemkem	112	12.9	3.3	22.5	90	19.4	8.9	29.9
Farta	138	13.5	1.9	25.1	106	17.0	5.6	28.3
Lay Gayint	126	6.6	0.8	12.4	111	15.5	0.3	30.8
Simada	162	11.3	5.8	16.7	73	1.3	0.0	3.0
Tach Gayint	138	7.1	0.0	15.4	87	9.0	0.0	18.2
**Zone**	1,309	17.4	13.0	21.7	982	21.4	16.5	26.4

Results from a cross-sectional survey in South Gondar zone, Amhara Regional State, Ethiopia in 2011.

* Estimates weighted according to selection probabilities and adjusted for correlation in the data due to clustering.

CI, confidence interval.

### Household-Level Water, Sanitation, and Hygiene

**Figure 4:**
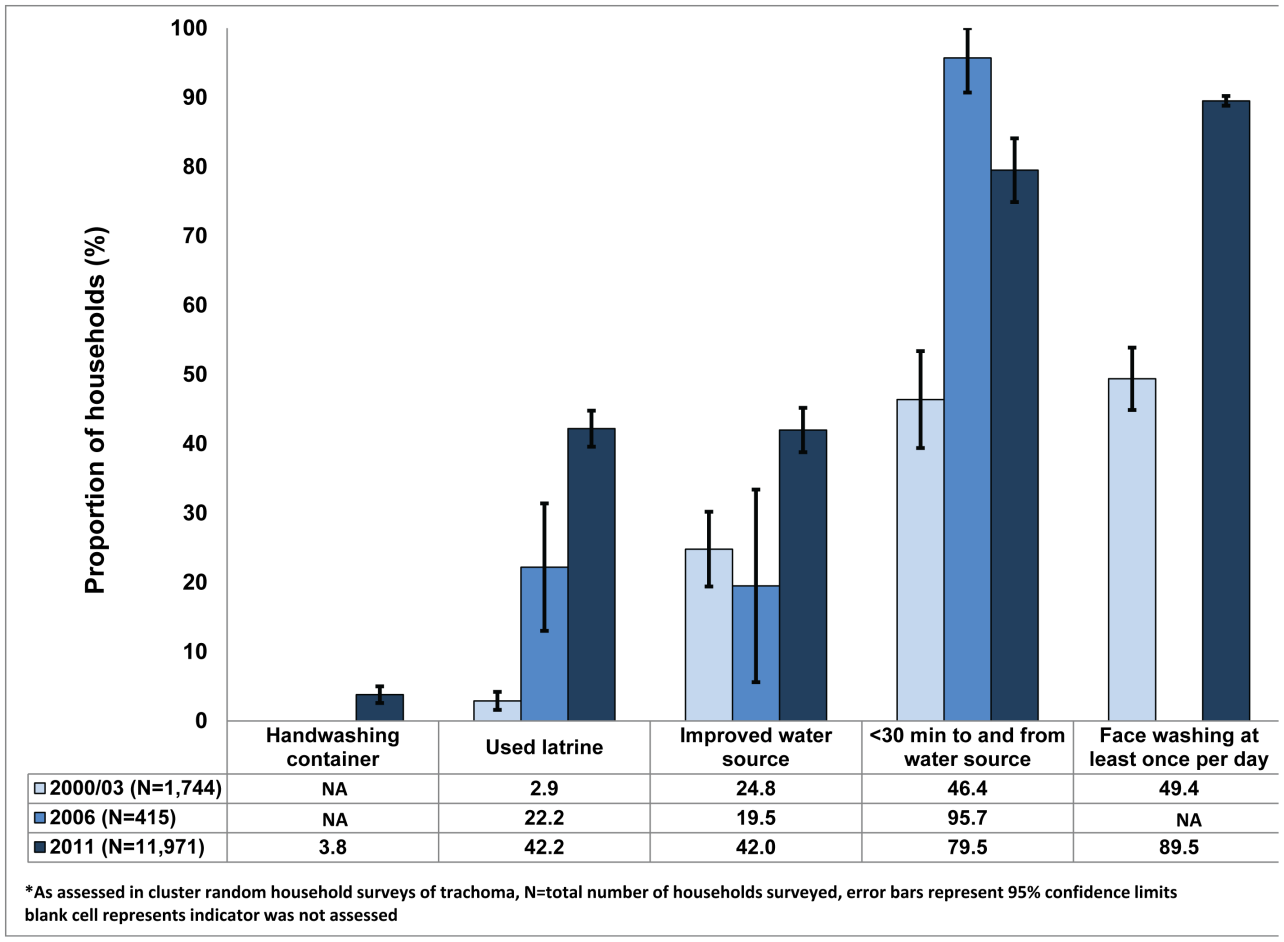
Proportion of households with basic sanitation and access to water in South Gondar, Amhara Region, Ethiopia in three surveys*.

A comparison of household-level indicators revealed statistically significant differences in household latrine ownership (Χ^2^ = 32.47, p<0.001), use of an improved source of water for drinking (Χ^2^ = 6.31, p = 0.012), reported access to water within 30 min collection time (Χ^2^ = 34.44, p<0.001), and reported frequency of washing faces of children under the age of 6 years (Χ^2^ = 23.28, p<0.001) compared to the baseline household surveys for trachoma done in 2000 and 2003 (Figure 4). In 3.8% (95% CI 2.6–5.0%) of households, the presence of a container outside of the latrine to hold water for washing hands was observed in 2011, but this indicator was not assessed in prior surveys. There has been a 14-fold increase in household latrine ownership, a 69.4% increase in reported household use of an improved water source, and a 71.3% increase in household access to water as defined by the reported round trip time of less than 30 min to collect water from the source. Among families with children younger than 6 years of age, the proportion reporting to wash the child's face at least once per day has increased by 81.2% since the implementation of the SAFE strategy.

### Reported Albendazole Coverage

The estimated drug coverage with albendazole is presented in Table 3. The proportion of children aged 2–6 years (preschool age) reported to have taken albendazole in the past year was 14.9% (95% CI 9.3–20.5%; range by *woreda* 0.8–33.0%) and 35.1% (95% CI 24.3–45.8%; range by *woreda* 10.3–68.4%) reported to have ever taken albendazole. The proportion of school-aged children reported to have ever taken albendazole was 33.2% (95% CI 22.9–43.5%; range by *woreda* 12.4–65.7%).

**Table 3:** Proportion* of children reporting to have taken albendazole in South Gondar, Ethiopia in 2011.

	Preschool-aged children (2–6 years)							School-aged children (7–15 years)			
		Taken within the past year			Ever taken				Ever taken		
*Woreda*	n	%	95% CI		%	95% CI		n	%	95% CI	
West Estie	148	8.8	1.0	16.7	17.3	4.3	30.2	88	16.0	3.1	28.8
Dera	130	31.9	17.1	46.7	68.4	41.9	94.8	110	63.1	35.6	90.6
Fogera	111	0.8	0.0	2.2	10.3	0.0	22.6	107	16.2	0.0	38.5
East Estie	114	12.4	2.9	22.0	22.0	9.2	34.9	84	12.4	1.6	23.1
Ebinat	118	33.0	4.5	61.4	42.4	14.3	70.6	106	28.8	2.6	55.0
Libokemkem	109	4.2	0.7	7.8	12.3	3.6	20.9	88	24.8	3.9	45.7
Farta	131	31.5	10.3	52.6	63.6	26.1	100.0	105	65.7	34.1	97.3
Lay Gayint	124	4.7	0.0	10.1	30.3	2.4	58.2	109	23.1	0.0	46.9
Simada	157	9.3	0.0	21.2	28.8	9.8	47.8	70	25.8	4.5	47.1
Tach Gayint	136	6.3	0.4	12.3	38.5	5.0	72.0	86	27.5	5.1	49.8
**Zone**	1,278	14.9	9.3	20.5	35.1	24.3	45.8	953	33.2	22.9	43.5

* Estimates weighted according to selection probabilities adjusted for correlation in the data due to clustering.

CI, confidence interval.

### Comparison with Historical Helminth Data

**Figure 5:**
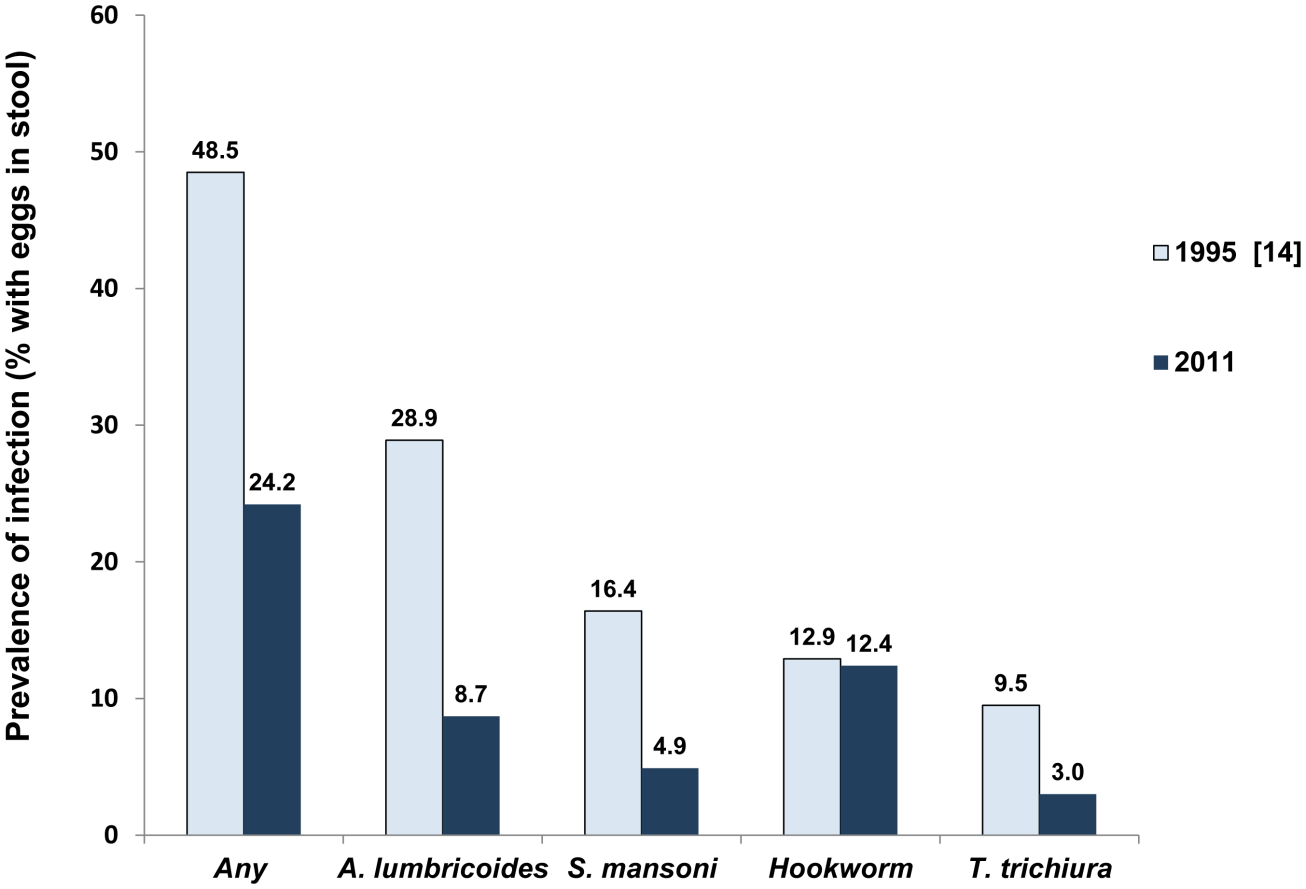
Prevalence of helminth infections among school-aged children in South Gondar (1995 and 2011).

The estimated prevalence of each, *A. lumbricoides*, *T trichiura* and *S. mansoni*, infection was considerably lower than reported in 1995 (Figure 5). The prevalence of hookworm infection was not different from the previous estimate. Table 4 presents a comparison of the historical survey to data in the current study, restricted to children aged 7–15 years both within only the six *woredas* represented in the 1995 study (column 2) and within all 10 *woredas* covered in 2011 (column 3). For each of the helminths compared, infections were identified in a smaller proportion of communities in the current survey than observed in 1995. *A. lumbricoides* was the only helminth infection for which more than 100 eggs were counted per specimen, representing a prevalence of 1.9% (95% CI 0.8–2.9%). Without counting all the eggs identified in those specimens, a classification as moderate or high intensity using the standardized eggs per gram of stool (EPG) thresholds frequently employed when using the Kato-Katz thick smear method is not possible [23]. Even after adjusting for the exact weight of stool preserved, all other infections identified would be classified as low intensity infections in contrast to the 1995 findings (Table 2).

**Table 4:** Comparison of helminth prevalence data in South Gondar 1995 and 2011.

Survey	Jemaneh (1995) [14]	Trachoma impact evaluation 2011*	Trachoma impact evaluation 2011
Sample population	School children	Children in community	Children in community
*Woredas* covered	6	same 6 as in 1995	all 10 rural *woredas*
Communities	22	60	99
Total children	2,279 (7–19 years of age)	617 (7–15 years of age)	982 (7–15 years of age)
Median age (years)	12	10	10
Mean age (years)	NA	11.0 (SD 2.4)	10.9 (SD 2.4)
Specimens per child	Single	Single	Single
Technique	Kato-Katz	Ether-concentration of SAF-fixed specimens	Ether concentration of SAF-fixed specimens

Clinical findings	Infection prevalence %	Positive schools	Intensity‡ >200 eggs/g	Prevalence** % (95% CI)	Positive *gotts*	Intensity‡ >100 eggs/g	Infection prevalence** % (95% CI)	Positive *gotts*	Intensity‡ >100 eggs/g
*S. mansoni*	16.4	63.6%	14.6	4.9 (0.5–9.2)	13.3%	0.0	3.6 (0.5–6.6)	16.2%	0.0
*A. lumbricoides*	28.9	90.9%	28.5	8.7 (4.8–12.6)	45.0%	0.9 (0.1–1.8)	10.8 (7.2–14.3)	66.7%	1.9 (0.8–2.9)
*T. trichiura*	9.5	86.4%	8.3	3.0 (1.1–4.8)	21.7%	0.0	3.2 (1.6–4.8)	35.4%	0.0
Hookworm	12.9	77.3%	12.1	12.4 (6.2–18.7)	43.3%	0.0	11.0 (6.5–15.5)	56.6%	0.0
Any above infection	48.5	100%	NA	24.2 (17.6–30.9)	73.3%	0.9 (0.1–1.8)	23.9 (18.8–29.0)	85.9%	1.9 (0.8–2.9)

* Data set limited to school-aged children from only the same six *woredas* included in 1995.

‡ Prevalence of intense infection measured as proportion of total sampled population presenting with >200 eggs per gram of feces (1995) or >100 eggs per gram of feces (2011).

** Estimates weighted according to selection probabilities adjusted for correlation in the data due to clustering.

## Discussion

Our study done in 2011 revealed a considerably different epidemiological portrait of soil-transmitted helminths and *S. mansoni* in South Gondar zone of central Ethiopia than the one painted in 1995. Indeed, the infection prevalence of compared helminths has declined substantially, except for hookworm, and infection intensities have concurrently declined for all the identified helminths. These changes have occurred in the context of the health extension program (HEP), the implementation of the SAFE strategy for the control of trachoma, and EOS. The HEP is a major undertaking since 2004 to provide access to preventive health services to the rural communities of Ethiopia and serves as the backbone of SAFE implementation in the communities [24].

The SAFE strategy was implemented by the Amhara National Regional State Health Bureau in pilot areas of South Gondar starting in 2003 and, by 2006, the program was operating at scale in all *woredas* due to simultaneous scale-up of HEP having in place at least one health extension worker in each *kebele* (village). In addition to the ongoing promotion of behavior change communication in 337 *kebeles* of South Gondar, a total of 339,913 household latrines have been reported to be constructed since pilot interventions in 2003 [South Gondar Zonal Health Department reports, unpublished data]. We have presented evidence (Figure 4) from a series of cross-sectional surveys indicating statistically significant improvements in reported hygiene behavior (e.g., washing faces of young children), use of an improved water source, improved access to water, and household-level access to basic sanitation (e.g., presence of a used latrine). If each of the 339,913 households, latrines reported to be constructed were first latrines of households. Hence, the corresponding latrine coverage should be as high as 72.6%, which is considerably higher than the 42.2% coverage identified in this study. There are several possible explanations for the discordance, which may contribute to the difference independently or in combination: health workers double-counted latrines or reported them as complete before they were, the reports from the districts were inflated to exaggerate progress, the collation of reports at district level was not accurate, a proportion of the new latrines reported were actually new replacements for households that already had one and therefore would not add to the numerator of households with a latrine, or the number of household units and population has grown significantly so as to increase the denominator – as previously highlighted to be a challenge to meeting the MDG 7c target [25]. Whatever the reasons, the discordance outlines the importance of periodic household surveys to serve as an independent monitor of the uptake of promoted interventions. Needless to say, despite improvements in access to sanitation (from 3% in 2003 to 42% in 2011), interventions are still needed, as more than half of households surveyed were without a toilet and using an unimproved source of water. While the presence of a water container for hand washing was observed outside a latrine in only a small proportion of households, some people are adopting a recently promoted health message even though there is a long way to go. These findings might explain the frequent intestinal protozoa infections identified. We have no background data to assess any change in intestinal protozoa infection prevalence, but presence of these infections suggests that the water being used for drinking is of poor quality. Whether contamination is occurring at the source, collection, or storage should be further investigated, so that adequate mitigation strategies can be implemented.

Albendazole was distributed to children aged 2–5 years every 6 months in EOS campaigns since 2004. At the national level, it has been reported that up to 9 million doses of albendazole have been distributed per round [16]. The program was originally targeted to children in *woredas* labeled as “food insecure”, but has since been expanded. Coverage surveys to evaluate the EOS have reported achievement of 93.8% of targeted children for albendazole in 2006 and 92.1% for vitamin A in 2008 [26]. Since 2009 in South Gondar, a cumulative 945,991 doses of albendazole have been distributed to approximately 213,000 preschool-aged children during such campaigns with a reported coverage of 100% (range by year 98.3–106%) [South Gondar Zonal Health Department, unpublished data]. The reported coverage figures are in sharp contrast to our survey estimates. Coverage estimates of mass drug administration (MDA) programs are commonly lower than administrative reports [27], [28]. At the most, only one out of every three preschool-aged children reported taking the drug within the past year. From Table 3, it is evident that the food insecure *woredas* were likely to be Dera, Ebinat, and Farta, which had the highest proportions of children in both age groups reporting ever having taken albendazole. If the distribution decisions were determined at a level below the *woreda*, then we may have misrepresented albendazole coverage by aggregating the results from non-targeted communities with targeted communities. Nonetheless, the findings confirm the importance of independently assessing MDA coverage with household surveys.

Our survey has some limitations. First, the prevalence estimates of helminths and intestinal protozoa are based on a small amount of stool from a single specimen. Helminth egg output varies from one day to another and within each stool specimen, hence it is probable that we have underestimated the ‘true’ prevalence, although less likely intense infections [29], [30]. However, this variation should be comparable to the single stool results presented by Jemenah in the mid-1990s which had the same limitation [14]. Second, we avoided calculating percent decreases in prevalence due to the different diagnostic techniques used in 1995 (fresh stool samples using the Kato-Katz technique [31]) and the current study (SAF-fixed stool samples subjected to an ether-concentration method [18], [20]). Using the Kato-Katz technique may have allowed a more direct comparison to the baseline data and a more precise measure of infection intensity, but it was not feasible given the logistical challenges posed by community-based surveys in the remote settings surveyed here. Our comparison of intensity of infection was limited, but in determining prevalence, fixing of stool samples in SAF and employing an ether-concentration method is as sensitive as the Kato-Katz technique [32]. Previous studies consistently revealed low sensitivity of the Kato-Katz technique in detecting hookworm infections, particularly those of low-intensity [33]–[35]. Third, we did not assess the cleanliness of the observed latrines. While improved sanitation is protective against soil-transmitted helminthiasis, a latrine with feces around the drop hole, in theory, may serve as a source of hookworm transmission [36], [37]. Fourth, our albendazole coverage estimates are subject to recall bias. However, we took steps to minimize recall bias by showing the albendazole tablets distributed during EOS campaigns and the most recent round of EOS was implemented less than one month prior to the survey. Additionally, other MDA participation studies reported that individuals are capable of recalling whether they have taken a drug during the distribution [38], [39]. Albendazole coverage, even in targeted *woredas*, was very low, which we feel provides stronger support to the hypothesis that improvements in F and E were largely responsible for the decline in helminth infection prevalence. However, this study was cross-sectional and therefore inherently has the inability to link causal associations with improvements in the sanitation due to SAFE and preventive chemotherapy due to unmeasured confounding factors. An alternative hypothesis is that the recorded, significant improvements in latrines, water access, and face washing have had minimal impact on intestinal parasites and the decline is due to secular variation. The two surveys compared were conducted nearly 16 years apart. We cannot rule out a secular decline in the prevalence and intensity of helminth infections, but a national survey of school-aged children in 2006 reported a prevalence of *A. lumbricoides* of 28.0% and of any soil-transmitted helminth of 37.7%, perhaps indicating that from 1995 to 2006 there may have been little change in prevalence due to secular variation in South Gondar [40]. Hence, further investigation of predictive factors of the observed infections is warranted.

This study demonstrates the feasibility and success of an integrated neglected tropical disease assessment for programmatic decision making. Despite the low intensity of identified helminth infections, infection with any of the helminths targeted for control was identified in over 80% of the communities surveyed. The *woreda*-level prevalence of any soil-transmitted helminth exceeded 20% in East Estie, West Estie, Dera, and Fogera *woredas*, and hence, according to WHO guidelines, warrants preventive chemotherapy targeting school-aged children [41]. Additionally, preventive chemotherapy using praziquantel against schistosomiasis is warranted in Fogera *woreda* and other communities where the proportion of children infected with *S. mansoni* was greater than 10%. Through this assessment, we were able also to identify several intestinal protozoa infections, some of which contribute to morbidity [42]. At the least, the high prevalence of these infections indicates contamination of water at the point of the source or use and warrants further investigation and setting-specific interventions. It also suggests that there is much more work to be done in improving water quality, hygiene, and sanitation in these mostly rural areas of Ethiopia.

While we cannot directly attribute the decline in helminth prevalence and intensity directly to the SAFE strategy, the documented increase in hygiene and sanitation offer both a biologically plausible and parsimonious explanation for the decline which is consistent with our understanding of the epidemiology of helminth and intestinal protozoa infections. Preventive chemotherapy in national helminth control programs has been shown to significantly reduce prevalence and intensity of helminth infections and has likely contributed to the observed decline [43]–[45]. However, without environmental changes, there is potential for rapid reinfection and continued transmission [46], [47]. Additionally, participation, defined as ever taking albendazole, among the targeted population as reported in this survey was much lower than administrative records suggest. Given the simultaneous scaling up of both F and E from the SAFE strategy and de-worming in EOP since 2006, one has to consider that there has been a synergistic effect of these ongoing interventions even though coverage (both household latrine ownership and preventive chemotherapy with albendazole) has been below target. There remain opportunities for integrated neglected tropical disease control throughout Ethiopia [48]. These results are encouraging and present a portrait of what might be expected within an integrated, multi-sectoral package of interventions for neglected tropical disease control.

## Supporting Information

Checklist S1 **STROBE checklist.**
(DOC)
